# 4,7-Phenanthrolinium perchlorate–5-methyl-1,2,4-triazolo[1,5-*a*]pyrimidin-7(4*H*)-one–water (1/1/2)

**DOI:** 10.1107/S1600536810002564

**Published:** 2010-01-27

**Authors:** Ana B. Caballero, Miguel Quirós, Juan M. Salas

**Affiliations:** a Departamento de Química Inorgánica, Facultad de Ciencias, Universidad de Granada, 18071 Granada, Spain

## Abstract

The asymmetric unit of the title compound, C_12_H_9_N_2_^+^·ClO_4_^−^·C_6_H_6_N_4_O·2H_2_O, contains a monoprotonated 4,7-phenanthrolinium (47phen) cation, a perchlorate anion balancing its charge, a neutral mol­ecule of 5-methyl-1,2,4-triazolo[1,5-*a*]pyrimidin-7(4*H*)-one (HmtpO) and two inter­stitial water mol­ecules. In the crystal structure, the acidic H atoms of 47phenH^+^ and HmtpO form strong hydrogen bonds with the water mol­ecules, which in turn act as hydrogen-bond donors, forming links between them and towards the carbonyl O atom of HmtpO, the non-protonated N atom of 47phen^+^ and one of the O atoms of the anion.

## Related literature

For other structures containing perchlorate and protonated 4,7-phenanthroline, see: Shang *et al.* (2006 ▶); Gillard *et al.* (1998 ▶). For other structures containing neutral and non-coordinated 5-methyl-1,2,4-triazolo[1,5-*a*]pyrimidin-7(4*H*)-one, see: Navarro *et al.* (1997 ▶); Salas *et al.* (1996 ▶).
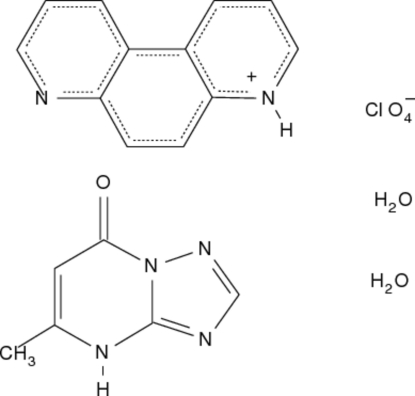


## Experimental

### 

#### Crystal data

C_12_H_9_N_2_^+^·ClO_4_^−^·C_6_H_6_N_4_O·2H_2_O
*M* *_r_* = 466.84
Monoclinic, 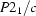

*a* = 8.6082 (8) Å
*b* = 14.7723 (14) Å
*c* = 16.8079 (17) Å
β = 104.609 (2)°
*V* = 2068.2 (3) Å^3^
*Z* = 4
Mo *K*α radiation
μ = 0.24 mm^−1^
*T* = 298 K
0.42 × 0.38 × 0.13 mm

#### Data collection

Bruker SMART APEX CCD diffractometer
Absorption correction: multi-scan (*SADABS*; Bruker, 1999 ▶) *T*_min_ = 0.764, *T*_max_ = 0.969
12883 measured reflections
4653 independent reflections
3687 reflections with *I* > 2σ(*I*)
*R*_int_ = 0.029

#### Refinement

*R*[*F* ^2^ > 2σ(*F* ^2^)] = 0.054
*wR*(*F* ^2^) = 0.152
*S* = 1.03
4653 reflections
302 parameters
4 restraints
H atoms treated by a mixture of independent and constrained refinement
Δρ_max_ = 0.40 e Å^−3^
Δρ_min_ = −0.37 e Å^−3^

### 

Data collection: *SMART* (Bruker, 1999 ▶); cell refinement: *SAINT* (Bruker, 1999 ▶); data reduction: *SAINT*; program(s) used to solve structure: *SHELXS97* (Sheldrick, 2008 ▶); program(s) used to refine structure: *SHELXL97* (Sheldrick, 2008 ▶); molecular graphics: *Xtal_GX* (Hall & du Boulay, 1997 ▶); software used to prepare material for publication: *SHELXL97*.

## Supplementary Material

Crystal structure: contains datablocks I, global. DOI: 10.1107/S1600536810002564/lh2981sup1.cif

Structure factors: contains datablocks I. DOI: 10.1107/S1600536810002564/lh2981Isup2.hkl

Additional supplementary materials:  crystallographic information; 3D view; checkCIF report

## Figures and Tables

**Table 1:** Hydrogen-bond geometry (Å, °)

*D*—H⋯*A*	*D*—H	H⋯*A*	*D*⋯*A*	*D*—H⋯*A*
N4—H4⋯O2*W*	0.86	1.89	2.743 (2)	173
N4*P*—H4*P*⋯O1*W*	0.86	1.84	2.699 (3)	175
O1*W*—H11*W*⋯O7^i^	0.82 (1)	1.96 (2)	2.733 (3)	158 (3)
O1*W*—H12*W*⋯O2*W*^ii^	0.82 (1)	2.10 (1)	2.913 (3)	173 (3)
O2*W*—H21*W*⋯O3^iii^	0.82 (1)	2.09 (1)	2.875 (3)	162 (3)
O2*W*—H22*W*⋯N7*P*^iv^	0.82 (1)	1.96 (1)	2.771 (3)	177 (3)

Symmetry codes: (i) 
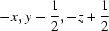
; (ii) 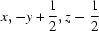; (iii) 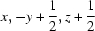; (iv) 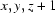.

**Fig. 1.**
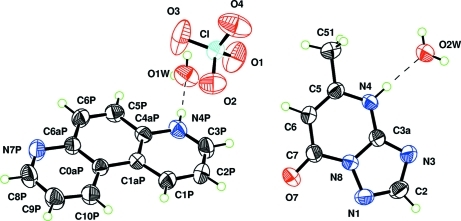
View of the asymmetric unit of the title compound with the displacement ellipsoids shown at the 50% probability level. Hydrogen bonds are shown as dashed lines.

## supplementary crystallographic information

### Comment

The title compound was obtained as a by-product when trying to synthesize a copper complex containing both heterocycles, as indicated in the preparation section. The formula of the compound is (47phenH)(HmtpO)(ClO_4_).2H_2_O (47phen = 4,7-phenanthroline and HmtpO = 5-methyl-1,2,4-triazolo[1,5-*a*]pyrimidine-7(4*H*)-one), which also correspond to the contents of the asymmetric unit which is shown in Figure 1. The geometrical parameters of both heterocycles do not significantly differ from other compounds with protonated 47phen (Shang *et al.*, 2006, Gillard *et al.*, 1998) or neutral HmtpO (Navarro *et al.*, 1997, Salas *et al.* 1996). The species are linked in the crystal mainly by hydrogen bonds, water molecules being the main actors of the H-bond network. One of the independent water molecules (O1W) accepts an H-bond from the extra proton of 47phen (N4P—H) and donates towards the carbonyl O-atom (O7) of the triazolopyrimidine moiety and towards the other water molecule (O2W). The later also accepts an H-bond from the acidic H-atom of HmtpO (N4—H) acting as donor for the perchlorate anion and for the non-protonated N atom of 47phen (N7P). This builds a two-dimensional hydrogen bond network, which includes, among other motifs, centrosymmetric (HmtpO)_2_(H_2_O)_4_ boxes, with both HmtpO molecules stacked with a separation of 3.4 Å and linked by two chains with two water molecules each, starting at N4P of one of the heterocycles and ending at O7 of the other: N4P—H···O1W—H···O2W—H···O7.

### Experimental

The compound was fortuitously obtained as a by-product when trying to synthesize a ternary complex of Cu(II) with 5-methyl-1,2,4-triazolo[1,5-*a*]pyrimidine-7(4*H*)-one (HmtpO) and 4,7-phenanthroline (47phen). An aqueous solution (10 ml.) of Cu(ClO_4_)_2_.6H_2_O (0,75 g, 2 mmol), another aqueous solution (20 ml.) of HmtpO (0,61 g, 4 mmol) and a ethanolic solution (10 ml.) of 47phen (0,73 g, 4 mmol) were mixed and the mixture was refluxed for 2 h, a green precipitate (a Cu-Hmtpo complex) appearing which was filtered off. The mother liquor was left to stand at room temperature for two weeks, when a mixture of green and pale yellow crystals was obtained, which was filtered off. It was possible to separate both types of crystals under a lens, the green crystals turning out to be a Cu-phen complex whereas the pale yellow ones are the title compound, the structure of which is presented in this article. Elemental analysis data for C_18_H_19_ClN_6_O_7_. % Found (Calc.): C 46.17 (46.31), H 4.52 (4.10), N 17.79 (18.00).

### Refinement

Hydrogen atoms of the organic moieties were idealized with distances to their parent atoms of 0.93 (C) or 0.86 (N) Å, the location of acidic (N—H) H atoms being obvious from previous ΔF maps. Free rotation was allowed for the methyl group. Water hydrogen atoms were easily located in ΔF maps and refined with restrained O—H distances (0.82 (1) Å). Displacement parameters of all H atoms were fixed at 1.2 times the *U*_eq_ of their parent atoms.

### Crystal data

**Table:**

C_12_H_9_N_2_^+^·ClO_4_^−^·C_6_H_6_N_4_O·2H_2_O	*F*(000) = 968
*M**_r_* = 466.84	*D*_x_ = 1.499 Mg m^−^^3^
Monoclinic, *P*2_1_/*c*	Mo *K*α radiation, λ = 0.71073 Å
Hall symbol: -P 2ybc	Cell parameters from 3841 reflections
*a* = 8.6082 (8) Å	θ = 2.4–24.6°
*b* = 14.7723 (14) Å	µ = 0.24 mm^−^^1^
*c* = 16.8079 (17) Å	*T* = 298 K
β = 104.609 (2)°	Irregular, pale yellow
*V* = 2068.2 (3) Å^3^	0.42 × 0.38 × 0.13 mm
*Z* = 4	

### Data collection

**Table:**

Bruker SMART APEX CCD diffractometer	4653 independent reflections
Radiation source: fine-focus sealed tube	3687 reflections with *I* > 2σ(*I*)
graphite	*R*_int_ = 0.029
Detector resolution: 8.26 pixels mm^-1^	θ_max_ = 28.3°, θ_min_ = 1.9°
φ and ω scans	*h* = −11→11
Absorption correction: multi-scan (*SADABS*; Bruker, 1999)	*k* = −18→16
*T*_min_ = 0.764, *T*_max_ = 0.969	*l* = −22→13
12883 measured reflections	

### Refinement

**Table:**

Refinement on *F*^2^	Primary atom site location: structure-invariant direct methods
Least-squares matrix: full	Secondary atom site location: difference Fourier map
*R*[*F*^2^ > 2σ(*F*^2^)] = 0.054	Hydrogen site location: mixed
*wR*(*F*^2^) = 0.152	H atoms treated by a mixture of independent and constrained refinement
*S* = 1.03	*w* = 1/[σ^2^(*F*_o_^2^) + (0.080*P*)^2^ + 0.7*P*] where *P* = (*F*_o_^2^ + 2*F*_c_^2^)/3
4653 reflections	(Δ/σ)_max_ = 0.001
302 parameters	Δρ_max_ = 0.40 e Å^−^^3^
4 restraints	Δρ_min_ = −0.37 e Å^−^^3^

### Special details

**Table:**

Geometry. All e.s.d.'s (except the e.s.d. in the dihedral angle between two l.s. planes) are estimated using the full covariance matrix. The cell e.s.d.'s are taken into account individually in the estimation of e.s.d.'s in distances, angles and torsion angles; correlations between e.s.d.'s in cell parameters are only used when they are defined by crystal symmetry. An approximate (isotropic) treatment of cell e.s.d.'s is used for estimating e.s.d.'s involving l.s. planes.
Refinement. Refinement of *F*^2^ against ALL reflections. The weighted *R*-factor *wR* and goodness of fit *S* are based on *F*^2^, conventional *R*-factors *R* are based on *F*, with *F* set to zero for negative *F*^2^. The threshold expression of *F*^2^ > σ(*F*^2^) is used only for calculating *R*-factors(gt) *etc*. and is not relevant to the choice of reflections for refinement. *R*-factors based on *F*^2^ are statistically about twice as large as those based on *F*, and *R*- factors based on ALL data will be even larger.

### Fractional atomic coordinates and isotropic or equivalent isotropic displacement parameters (Å^2^)

**Table:**

	*x*	*y*	*z*	*U*_iso_*/*U*_eq_	
Cl	0.45313 (6)	0.33264 (4)	0.31768 (3)	0.04631 (18)	
O1	0.3034 (3)	0.31480 (17)	0.33511 (15)	0.0894 (7)	
O2	0.4656 (3)	0.42667 (14)	0.30285 (15)	0.0838 (6)	
O3	0.4615 (4)	0.28347 (18)	0.24717 (16)	0.1138 (10)	
O4	0.5784 (3)	0.30880 (19)	0.38506 (17)	0.1111 (9)	
N1	0.0430 (2)	0.68226 (12)	0.53673 (12)	0.0482 (5)	
C2	0.0189 (3)	0.67004 (16)	0.61003 (16)	0.0521 (6)	
H2	−0.0348	0.7130	0.6336	0.063*	
N3	0.0760 (2)	0.59263 (13)	0.65035 (12)	0.0470 (4)	
C3A	0.1429 (2)	0.55371 (13)	0.59692 (12)	0.0372 (4)	
N4	0.2222 (2)	0.47442 (11)	0.60339 (10)	0.0390 (4)	
H4	0.2287	0.4404	0.6456	0.047*	
C5	0.2917 (3)	0.44833 (14)	0.54280 (13)	0.0416 (5)	
C51	0.3840 (3)	0.36204 (17)	0.55767 (17)	0.0591 (6)	
H51	0.4143	0.3444	0.5087	0.071*	
H52	0.3184	0.3156	0.5725	0.071*	
H53	0.4786	0.3704	0.6016	0.071*	
C6	0.2757 (3)	0.49988 (15)	0.47456 (13)	0.0456 (5)	
H6	0.3213	0.4791	0.4334	0.055*	
C7	0.1924 (3)	0.58373 (15)	0.46251 (13)	0.0438 (5)	
O7	0.1771 (2)	0.63513 (13)	0.40383 (10)	0.0629 (5)	
N8	0.1250 (2)	0.60466 (11)	0.52801 (10)	0.0375 (4)	
C1P	0.2195 (3)	0.60343 (16)	0.13977 (14)	0.0509 (6)	
H1P	0.2555	0.6615	0.1325	0.061*	
C1AP	0.2186 (2)	0.53636 (14)	0.08008 (12)	0.0388 (4)	
C2P	0.1672 (3)	0.58385 (19)	0.20817 (15)	0.0608 (7)	
H2P	0.1682	0.6283	0.2475	0.073*	
C3P	0.1126 (3)	0.49785 (19)	0.21863 (15)	0.0565 (6)	
H3P	0.0758	0.4847	0.2648	0.068*	
N4P	0.1128 (2)	0.43452 (14)	0.16298 (11)	0.0480 (5)	
H4P	0.0784	0.3814	0.1707	0.058*	
C4AP	0.1650 (2)	0.44961 (14)	0.09408 (13)	0.0401 (5)	
C5P	0.1638 (3)	0.37718 (15)	0.03805 (14)	0.0488 (5)	
H5P	0.1270	0.3202	0.0482	0.059*	
C6P	0.2157 (3)	0.39210 (15)	−0.02966 (14)	0.0501 (5)	
H6P	0.2193	0.3440	−0.0648	0.060*	
C6AP	0.2661 (2)	0.47963 (15)	−0.04881 (13)	0.0423 (5)	
N7P	0.3086 (3)	0.48934 (14)	−0.12090 (12)	0.0548 (5)	
C8P	0.3516 (3)	0.57049 (19)	−0.14009 (16)	0.0611 (7)	
H8P	0.3800	0.5776	−0.1896	0.073*	
C9P	0.3551 (3)	0.64575 (18)	−0.09078 (16)	0.0617 (7)	
H9P	0.3864	0.7016	−0.1071	0.074*	
C10P	0.3139 (3)	0.63783 (17)	−0.01789 (15)	0.0539 (6)	
H10P	0.3155	0.6880	0.0157	0.065*	
C0AP	0.2681 (2)	0.55214 (14)	0.00543 (13)	0.0397 (5)	
O1W	0.0223 (2)	0.26333 (13)	0.18493 (13)	0.0679 (5)	
H11W	−0.045 (3)	0.237 (2)	0.1499 (15)	0.081*	
H12W	0.095 (3)	0.2279 (18)	0.2039 (19)	0.081*	
O2W	0.2656 (3)	0.37456 (12)	0.74489 (11)	0.0640 (5)	
H21W	0.335 (3)	0.3355 (16)	0.7542 (19)	0.077*	
H22W	0.275 (4)	0.4077 (17)	0.7846 (13)	0.077*	

### Atomic displacement parameters (Å^2^)

**Table:**

	*U*^11^	*U*^22^	*U*^33^	*U*^12^	*U*^13^	*U*^23^
Cl	0.0445 (3)	0.0470 (3)	0.0486 (3)	−0.0056 (2)	0.0139 (2)	−0.0066 (2)
O1	0.0712 (13)	0.1078 (18)	0.1028 (18)	−0.0295 (12)	0.0472 (13)	−0.0301 (14)
O2	0.0952 (16)	0.0500 (11)	0.1048 (17)	−0.0044 (10)	0.0228 (14)	0.0031 (11)
O3	0.171 (3)	0.0986 (18)	0.1052 (18)	−0.0598 (18)	0.0961 (19)	−0.0507 (15)
O4	0.0844 (17)	0.1027 (19)	0.120 (2)	0.0050 (14)	−0.0229 (15)	0.0294 (16)
N1	0.0501 (11)	0.0397 (10)	0.0580 (12)	0.0076 (8)	0.0193 (9)	0.0062 (8)
C2	0.0545 (13)	0.0445 (12)	0.0642 (15)	0.0067 (10)	0.0273 (12)	−0.0001 (11)
N3	0.0546 (11)	0.0445 (10)	0.0475 (11)	0.0028 (8)	0.0232 (9)	0.0028 (8)
C3A	0.0364 (10)	0.0377 (10)	0.0383 (10)	−0.0050 (8)	0.0109 (8)	0.0017 (8)
N4	0.0458 (9)	0.0350 (9)	0.0354 (9)	0.0014 (7)	0.0089 (7)	0.0048 (7)
C5	0.0440 (11)	0.0378 (11)	0.0431 (11)	0.0009 (9)	0.0110 (9)	−0.0030 (9)
C51	0.0713 (16)	0.0464 (13)	0.0624 (15)	0.0156 (12)	0.0223 (13)	0.0041 (12)
C6	0.0550 (13)	0.0458 (12)	0.0388 (11)	0.0042 (10)	0.0174 (10)	−0.0018 (9)
C7	0.0468 (11)	0.0483 (12)	0.0363 (11)	0.0011 (9)	0.0109 (9)	0.0023 (9)
O7	0.0826 (13)	0.0654 (11)	0.0457 (9)	0.0198 (9)	0.0254 (9)	0.0208 (8)
N8	0.0400 (9)	0.0337 (8)	0.0385 (9)	0.0012 (7)	0.0094 (7)	0.0034 (7)
C1P	0.0661 (15)	0.0418 (12)	0.0479 (13)	0.0001 (11)	0.0200 (11)	−0.0025 (10)
C1AP	0.0404 (10)	0.0384 (11)	0.0378 (11)	0.0008 (8)	0.0101 (9)	0.0010 (8)
C2P	0.0820 (18)	0.0600 (15)	0.0460 (13)	0.0092 (13)	0.0266 (13)	−0.0063 (12)
C3P	0.0645 (15)	0.0705 (17)	0.0407 (12)	0.0067 (13)	0.0250 (11)	0.0086 (12)
N4P	0.0493 (10)	0.0505 (11)	0.0458 (11)	−0.0016 (8)	0.0153 (8)	0.0108 (9)
C4AP	0.0383 (10)	0.0419 (11)	0.0396 (11)	0.0020 (8)	0.0090 (9)	0.0051 (9)
C5P	0.0572 (13)	0.0363 (11)	0.0530 (13)	−0.0077 (10)	0.0140 (11)	0.0004 (10)
C6P	0.0644 (14)	0.0394 (12)	0.0474 (13)	−0.0040 (10)	0.0157 (11)	−0.0078 (10)
C6AP	0.0454 (11)	0.0429 (11)	0.0396 (11)	−0.0017 (9)	0.0127 (9)	−0.0007 (9)
N7P	0.0691 (13)	0.0574 (12)	0.0421 (11)	−0.0034 (10)	0.0217 (10)	−0.0055 (9)
C8P	0.0750 (17)	0.0690 (17)	0.0456 (13)	−0.0101 (14)	0.0271 (13)	0.0053 (12)
C9P	0.0794 (18)	0.0540 (14)	0.0570 (15)	−0.0140 (13)	0.0271 (14)	0.0073 (12)
C10P	0.0715 (16)	0.0418 (12)	0.0513 (13)	−0.0093 (11)	0.0210 (12)	−0.0009 (10)
C0AP	0.0405 (10)	0.0399 (11)	0.0390 (11)	−0.0018 (8)	0.0106 (9)	−0.0001 (9)
O1W	0.0710 (13)	0.0532 (11)	0.0702 (13)	−0.0163 (9)	0.0007 (10)	0.0043 (9)
O2W	0.1006 (15)	0.0453 (10)	0.0443 (10)	0.0124 (10)	0.0149 (10)	0.0037 (8)

### Geometric parameters (Å, °)

**Table:**

Cl—O4	1.398 (2)	C1AP—C0AP	1.442 (3)
Cl—O3	1.407 (2)	C2P—C3P	1.381 (4)
Cl—O1	1.416 (2)	C2P—H2P	0.9300
Cl—O2	1.420 (2)	C3P—N4P	1.323 (3)
N1—C2	1.313 (3)	C3P—H3P	0.9300
N1—N8	1.374 (2)	N4P—C4AP	1.362 (3)
C2—N3	1.357 (3)	N4P—H4P	0.8600
C2—H2	0.9300	C4AP—C5P	1.424 (3)
N3—C3A	1.315 (3)	C5P—C6P	1.341 (3)
C3A—N4	1.346 (3)	C5P—H5P	0.9300
C3A—N8	1.357 (3)	C6P—C6AP	1.426 (3)
N4—C5	1.361 (3)	C6P—H6P	0.9300
N4—H4	0.8600	C6AP—N7P	1.359 (3)
C5—C6	1.354 (3)	C6AP—C0AP	1.404 (3)
C5—C51	1.489 (3)	N7P—C8P	1.318 (3)
C51—H51	0.9600	C8P—C9P	1.382 (4)
C51—H52	0.9600	C8P—H8P	0.9300
C51—H53	0.9600	C9P—C10P	1.364 (3)
C6—C7	1.420 (3)	C9P—H9P	0.9300
C6—H6	0.9300	C10P—C0AP	1.411 (3)
C7—O7	1.225 (3)	C10P—H10P	0.9300
C7—N8	1.402 (3)	O1W—H11W	0.816 (10)
C1P—C2P	1.367 (3)	O1W—H12W	0.819 (10)
C1P—C1AP	1.409 (3)	O2W—H21W	0.818 (10)
C1P—H1P	0.9300	O2W—H22W	0.816 (10)
C1AP—C4AP	1.402 (3)		
			
O4—Cl—O3	111.1 (2)	C4AP—C1AP—C0AP	118.41 (19)
O4—Cl—O1	110.00 (17)	C1P—C1AP—C0AP	123.79 (19)
O3—Cl—O1	108.70 (14)	C1P—C2P—C3P	119.8 (2)
O4—Cl—O2	108.15 (15)	C1P—C2P—H2P	120.1
O3—Cl—O2	109.56 (15)	C3P—C2P—H2P	120.1
O1—Cl—O2	109.35 (15)	N4P—C3P—C2P	119.9 (2)
C2—N1—N8	101.11 (17)	N4P—C3P—H3P	120.3
N1—C2—N3	117.5 (2)	C2P—C3P—H3P	119.8
N1—C2—H2	121.3	C3P—N4P—C4AP	123.0 (2)
N3—C2—H2	121.2	C3P—N4P—H4P	118.5
C3A—N3—C2	101.16 (18)	C4AP—N4P—H4P	118.5
N3—C3A—N4	128.72 (19)	N4P—C4AP—C1AP	119.04 (19)
N3—C3A—N8	111.43 (18)	N4P—C4AP—C5P	119.39 (19)
N4—C3A—N8	119.85 (17)	C1AP—C4AP—C5P	121.57 (19)
C3A—N4—C5	119.71 (17)	C6P—C5P—C4AP	119.3 (2)
C3A—N4—H4	120.1	C6P—C5P—H5P	120.4
C5—N4—H4	120.2	C4AP—C5P—H5P	120.3
C6—C5—N4	120.29 (19)	C5P—C6P—C6AP	121.7 (2)
C6—C5—C51	124.0 (2)	C5P—C6P—H6P	119.0
N4—C5—C51	115.68 (19)	C6AP—C6P—H6P	119.2
C5—C51—H51	109.7	N7P—C6AP—C0AP	122.4 (2)
C5—C51—H52	109.4	N7P—C6AP—C6P	117.6 (2)
H51—C51—H52	109.5	C0AP—C6AP—C6P	119.99 (19)
C5—C51—H53	109.4	C8P—N7P—C6AP	118.0 (2)
H51—C51—H53	109.5	N7P—C8P—C9P	123.3 (2)
H52—C51—H53	109.5	N7P—C8P—H8P	118.3
C5—C6—C7	123.46 (19)	C9P—C8P—H8P	118.3
C5—C6—H6	118.2	C10P—C9P—C8P	119.9 (2)
C7—C6—H6	118.3	C10P—C9P—H9P	120.1
O7—C7—N8	120.9 (2)	C8P—C9P—H9P	120.0
O7—C7—C6	127.1 (2)	C9P—C10P—C0AP	118.7 (2)
N8—C7—C6	111.98 (18)	C9P—C10P—H10P	120.6
C3A—N8—N1	108.82 (16)	C0AP—C10P—H10P	120.6
C3A—N8—C7	124.57 (17)	C6AP—C0AP—C10P	117.58 (19)
N1—N8—C7	126.40 (17)	C6AP—C0AP—C1AP	118.92 (19)
C2P—C1P—C1AP	120.4 (2)	C10P—C0AP—C1AP	123.5 (2)
C2P—C1P—H1P	119.8	H11W—O1W—H12W	108 (3)
C1AP—C1P—H1P	119.8	H21W—O2W—H22W	111 (3)
C4AP—C1AP—C1P	117.80 (19)		

### Hydrogen-bond geometry (Å, °)

**Table:**

*D*—H···*A*	*D*—H	H···*A*	*D*···*A*	*D*—H···*A*
N4—H4···O2W	0.86	1.89	2.743 (2)	173
N4P—H4P···O1W	0.86	1.84	2.699 (3)	175
O1W—H11W···O7^i^	0.82 (1)	1.96 (2)	2.733 (3)	158 (3)
O1W—H12W···O2W^ii^	0.82 (1)	2.10 (1)	2.913 (3)	173 (3)
O2W—H21W···O3^iii^	0.82 (1)	2.09 (1)	2.875 (3)	162 (3)
O2W—H22W···N7P^iv^	0.82 (1)	1.96 (1)	2.771 (3)	177 (3)

Symmetry codes: (i) −*x*, *y*−1/2, −*z*+1/2; (ii) *x*, −*y*+1/2, *z*−1/2; (iii) *x*, −*y*+1/2, *z*+1/2; (iv) *x*, *y*, *z*+1.
